# Epidemiology and economic loss of fasciolosis and dicrocoeliosis in Arak, Iran

**DOI:** 10.14202/vetworld.2018.1648-1655

**Published:** 2018-12-10

**Authors:** Mohsen Arbabi, Elnaz Nezami, Hossein Hooshyar, Mahdi Delavari

**Affiliations:** Department of Medical Parasitology and Mycology, School of Medicine, Kashan University of Medical Sciences, Kashan, Iran

**Keywords:** dicrocoeliosis, economic loss, epidemiology, fasciolosis, Iran, slaughtered animal

## Abstract

**Aim:**

Fasciolosis and dicrocoeliosis are important parasitic diseases worldwide, causing significant financial losses due to decrease in production and viscera condemnation in animals. We performed the current research to assess the epidemiology of these infections and determine their significance from an economic perspective in Arak, Iran.

**Materials and Methods:**

In total, we evaluated 118,463 sheep, 207,652 goats, and 43,675 cattle through necropsic analysis at the slaughterhouses. The average weight of sheep, goat, and cattle liver was 1000, 900, and 5000 g, respectively. The average price of liver in the market was 8 USD/kg. Moreover, the elimination of fundamental nutrients and vitamins was evaluated in infected livers. The prevalence of fasciolosis and dicrocoeliosis was determined. Analysis of variance test was applied for the statistical analysis, and the significance level was <0.05.

**Results:**

In total, *Fasciola hepatica* and *Dicrocoelium dendriticum* were detected in 0.56% (confidence interval CI, 0.54-0.59) and 0.77% (CI, 0.75-0.81) of the animals, respectively (p=0.1). The annual economic loss attributed to fasciolosis and dicrocoeliosis was 26698.4 and 30479.2 USD, respectively. The total economic loss was 10,880, 9079.2, and 10,520 dollars in sheep, cattle, and goats, respectively. On the other hand, financial loss resulting from fasciolosis was 7160, 6098.4, and 13,440 dollars in sheep, goats, and cattle, respectively. In addition, economic loss due to dicroceliasis was 10,880, 9079.2, and 10,520 dollars, respectively.

**Conclusion:**

Overall, fasciolosis and dicrocoeliosis in Iran always remain common in sheep, goats, and cattle that afford major economic loss of all the country also exist in Arak province. The present study could provide basic information for further examination of liver fluke infections in Iran.

## Introduction

Farm animals play a significant role in human nutrition and socioeconomic evolution and development. Animal products such as milk and meat are a significant source of micronutrients, energy, and protein supply provided approximately 15% of energy and 30% of protein worldwide. In developing countries, with the increase in the number of residents and income growth, the use of animal protein per capita, with the global consumption of meat, is projected to increase by nearly 73% by 2050 [1]. Planning to these ensuring needs, as a major mission, leads to increased productivity of the production system, pasture management, food chains, and markets, and ultimately, and animal health [2]. Infectious diseases have been a serious threat to animal health and productivity in developing countries. A significant proportion of diseases affect the safety of food supplies, in addition to or instead of, their effect on the volume and quality of food products. Parasitological diseases, including those caused by trematodes, have widely differing effects on meat, milk, and fiber production and many new technologies have been developed to prevent or treat them [3,4]. Trematode infections such as dicrocoeliosis and fasciolosis in farm animals are an important parasitic disease cause serious economic losses to livestock worldwide. *Fasciola* species and *Dicrocoelium dendriticum* are recognized as the most common liver flukes through many regions of Asia including Iran [5-7]. These helminths are food-borne trematode zoonosis in ruminant and should be regarded as serious animal health problems in many urban and rural of the area, due to the rising number of infections in humans [8-10]. Goats, cattle, and sheep are the major definitive hosts of liver flukes. Nevertheless, in some cases, humans might act as incidental hosts and fasciolosis is recognized as emerging disease [11]. Fasciolosis due to *Fasciola hepatica* has a broad geographical distribution as a plant-borne zoonotic disease and occurs in >51 countries, especially where sheep and cattle are reared [12,13]. Trematode infections cause serious animal’s health problem and economic losses to livestock in many rural and urban areas of the world. The global economic burden of fasciolosis causing significant financial losses alone due to mortality, morbidity, decrease in production and growth rate, condemnations of the liver, and increased susceptibility to secondary infections and the expense of control measures in animal farming is considerable and is exceeding to 3 billion dollars each year for agricultural populations and producers [4,10,14-17].

Moreover, the infection has been reported in over 300 million cattle, as well as 250 million sheep around the world [18]. In fact, such losses can impose both direct and indirect losses. Host mortality and liver condemnation for the direct burdens of fasciolosis and dicrocoeliosis are significant.

On the other hand, indirect losses include reduced product quality, decreased rate of production, slow development, fertility impairment, and increased costs of stock replacement, and anthelmintic treatments [19-21]. In industrial countries, the prevalence of *F. hepatica* infection can reach up to 77%, and losses due to fasciolosis in some areas are >70.67 ­million pounds a year [9,22]. There is insufficient literature on the economic impact of dicrocoeliosis and fasciolosis in Iran. Therefore, it is necessary to perform research on the financial burdens of fasciolosis and dicrocoeliosis in this region. Fasciolosis, with a prevalence of 30-90%, is the most prevalent infection among cattle in tropical regions [18]. Considering its economic significance, fasciolosis has recently reemerged as a prevalent zoonotic disease, affecting different populations. At present, the infection has been reported as an important zoonosis for public health with up to 2.4-17 ­million people around the world; moreover, 180 million individuals are exposed to the risk [18,23]. Access to healthy sources of protein is necessary due to population growth. In different communities, the livestock provides the necessary proteins for people. Despite great progress in health-care provision and disease prevention, economic loss, resulting from food-borne liver fluke diseases, is still a major problem. According to statistics, the amount of financial loss is considerably every year. In general, financial losses arise from treatment costs, preventive measures for disease transmission, increased livestock mortality, and disposal of livestock [10]. Prevalence of fasciolosis in 13 Asian countries indicates with the highest range in cattle (0.71-69.2%) and lowest in goat (0.0-47.0%) [10]. Among Asian countries, Iran is one of the most important foci of the disease, and annually highest reports are recorded from the various provinces of this country [24]. In fact, both *F. ­hepatica* and *Fasciola gigantica* species are endemic to northern and western regions of Iran [24–26]. Reports show that the prevalence has been reported in sheep (0.35-31.25%), goat (0.20-4.4%), and cattle (0.71-81.5%), there are significant seasonal differences in the prevalence of *Fasciola* spp. [7,27-29].

The financial losses due to the condemnation of livers in Asian countries were calculated to be until 17.02 million USD annually [10]. Dicrocoeliosis is a hepatic parasitic infection affecting grazing ruminants and humans; however, it is found to be less severe than fasciolosis. The financial burden due to this infection is mainly attributed to liver condemnation [30]. A nationwide review of *Dicrocoelium* infection in slaughterhouses showed a prevalence of at least 0.1% up to 29.32% in Iran. The highest prevalence belongs to East Azerbaijan Province in northwestern, and the lowest belongs to Fars Province in the south of Iran [28,31,32].

Overall, scant research has focused on the economic loss and its public health significance of fasciolosis and dicrocoeliosis at national and regional levels; in fact, there is no adequate information on this region. So, we aimed to, determine the prevalence of fasciolosis and dicrocoeliosis in ruminant and, evaluated economic loss caused by the condemnation of livers at abattoirs of Arak in the center of Iran.

## Materials and Methods

### Ethical approval

The project was found to be in accordance with the ethical principles and standards for conducting medical research. This project was approved by Research Ethics Committee, Faculty of Medicine of Kashan University of Medical Sciences, Iran.

### Study area

**Figure-1:**
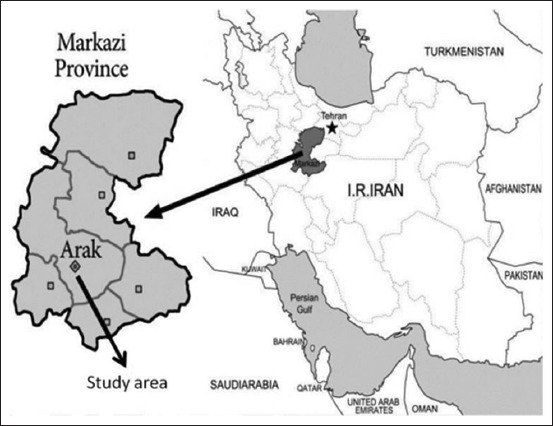
Map of study area.

Arak is a city of Markazi Province, Iran (Figure-1). It is located 34.09 latitude and 49.69 longitudes, and it is situated at elevation of 1750 meters above sea level. Arak has a Mediterranean continental climate that is, in general, relatively cold and dry. The average rainfall is around 350mm, and the annual relative humidity is 46%. Arak has a population of 503,647 making it the biggest city in Markazi province.

### Study design

In this cross-sectional study, data were collected during 2013-2016 to evaluate the prevalence of infection in the abattoirs of Arak and determine direct economic losses, resulting from liver condemnation through postmortem analysis. Through visualization and palpation, the liver and bile duct were examined.

### Study animals

Overall, 369,790 slaughtered sheep, goats, and cattle were recorded during necropsic analysis. *Fasciola* and *Dicrocoelium* parasites were evaluated in the removed liver and gallbladder. The species of parasites were identified, using the morphological characterization of *F. hepatica*, *F. gigantica*, and *D. dendriticum*.

### Estimation of economic loss

To determine the annual financial loss, the average number of slaughtered animals (per year in the abattoirs) was multiplied by the prevalence of infection in the current study and the mean price of liver in the region. We summarized the results for each year to determine the number of infected livers in animals. Using the available information, economic loss was estimated during 2013-2016. The total quantities were converted to US dollar. Moreover, direct economic loss imposed by fasciolosis and dicrocoeliosis was measured as follows:


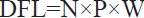


In this equation, DFL denotes direct financial loss, N represents the number of condemned livers, P is the average price of liver ($/kg), and W denotes the average weight of liver (kg). The average weight of the liver was measured by weighing 118,463 sheep, 207,652 goats, and 43,675 cattle livers. Furthermore, the average weight was measured to be 1000, 900, and 5000 g in sheep, goats, and cattle, respectively. According to interviews with local butchers, the average price of liver was 250,000 Rials/kg (8 dollars).

### Estimation of nutritional value

Minerals and vitamins were measured in each 100 g of liver, according to the protocol proposed by the United States Department of Agriculture [33].

### Statistical analysis

The data was processed by Microsoft Excel 2017 and IBM SPSS Statistics (Ver. 16, US) for determination of significant difference between variables. To evaluate the prevalence of fasciolosis and dicrocoeliosis, the number of animals infected with *Fasciola* and *Dicrocoelium* species was calculated (95% confidence interval). The number of infected cases was divided by the total number of slaughtered animals and multiplied by 100. Furthermore, for the comparison of fasciolosis and dicrocoeliosis in terms of prevalence, Analysis of variance test was carried out used to compare the prevalence of fasciolosis and dicrocoeliosis in different animals. Chi-squared test was used to analyze the effect of the season on the prevalence of the disease. The significance level was considered when the probability value p<0.05.

## Results

Overall, 369,790 slaughtered animals (118,463 sheep, 207,652 goats, and 43,675 cattle) were slaughtered and evaluated for *Fasciola* and *Dicrocoelium* species at Arak abattoirs, of which 2105 (0.56%) and 2884 (0.77%) were infected, respectively. The prevalence of *Fasciola* infection was 0.75%, 0.42%, and 0.76% in sheep, goats, and cattle, while the prevalence of *Dicrocoelium* infection was 1.14%, 0.60%, and 0.60%, respectively. However, the difference in prevalence rates of fasciolosis (p=0.21) and dicrocoeliosis (p=0.16) was not significant in various hosts (Table-1).

**Table-1:** The prevalence of condemned livers resulting from *Fasciola hepatica, Fasciola gigantica*, and *Dicrocoelium dendriticum* at slaughterhouses of Arak, Iran.

Host	Examined number	Fasciolosis		95% CI	Dicrocoeliosis		95% CI
	
		Infected liver number	Prevalence %		Infected liver number	Prevalence %	
Sheep	118,463	895	0.75	0.7-0.8	1360	1.14	1.08-1.2
Goat	207,652	874	0.42	0.39-0.44	1261	0.6	0.56-0.64
Cattle	43,675	336	0.76	0.68-0.84	263	0.6	0.52-0.67
Total	369,790	2105	0.56	0.54-0.59	2884	0.77	0.75-0.81
Difference between hosts		p=0.21		-	p=0.16		-

CI=Confidence interval

Furthermore, the prevalence of fasciolosis and dicrocoeliosis was evaluated during 4 seasons, as shown in Table-2. The prevalence of *Fasciola* infection in all hosts was the highest during winter, and the difference was significant (p=0.04). However, no seasonal trend was found in the prevalence of dicrocoeliosis, and differences were not of significance (p>0.1).

**Table-2:** Seasonal prevalence of liver condemnation resulting from *Fasciola hepatica, Fasciola gigantica*, and *Dicrocoelium dendriticum* in sheep, goats, and cattle at abattoirs of Arak, Iran.

Season	Slaughtered number			Sheep infected number %		Goat infected number %		Cattle infected number %	
			
	Sheep	Goat	Cattle	Fasciolosis	Dicrocoeliosis	Fasciolosis	Dicrocoeliosis	Fasciolosis	Dicrocoeliosis
Spring	25,265	60012	11351	183 (0.72)	225 (0.89)	233 (0.38)	203 (0.33)	63 (0.55)	47 (0.041)
Summer	36,489	63123	12931	231 (0.63)	418 (1.14)	258 (0.40)	433 (0.68)	92 (0.71)	88 (0.68)
Autumn	42,379	67407	9065	206 (0.48)	398 (0.93)	187 (0.27)	287 (0.42)	52 (0.57)	60 (0.66)
Winter	14,330	17110	10328	275 (1.91)	319 (2.22)	196 (1.14)	338 (1.97)	129 (1.24)	68 (0.65)
Total	118,463	207652	43675	895 (0.75)	1360 (1.14)	874 (0.42)	1261 (0.60)	336 (0.76)	263 (0.60)

The evaluation of economic impacts due to the *Fasciola* infection in the area study shows alarming values: 895 kg of sheep livers, 762 kg of goat livers, and 1680 kg of cattle livers were condemned. In addition, 1360 kg of sheep livers, 1135 kg of goat livers, and 1315 kg of cattle livers infected with dicrocoeliosis. Based on the analysis of market prices in Arak, the average price of 1 kg of liver was reported to be 8 dollars. The average annual loss of meat and offals, resulting from *Fasciola* and *Dicrocoelium* infections, was estimated at 26698.4 and 30479.2 USD, respectively. Overall, 7160, 6098.4, and 13,440 USD were lost due to fasciolosis in sheep, goats, and cattle, respectively. In addition, economic loss due to *Dicrocoelium* infection was 10,880, 9079.2, and 10,520 USD in sheep, goats, and cattle, respectively (Table-3).

**Table-3:** The annual financial loss resulting from *Fasciola hepatica*, *Fasciola gigantica*, and *Dicrocoelium dendriticum* in slaughtered animals.

Host	Examined number	Fasciolosis	Economic losses (USD)	Dicrocoeliosis	Economic losses (USD)
	
		Infected liver number		Infected liver number	
Sheep	118,463	895	7160	1360	10880
Goat	207,652	874	6098.4	1261	9079.2
Cattle	43,675	336	13440	263	10520
Total	369,790	2105	26698.4	2884	30479.2

Tables-4 and 5 present the analysis of macronutrient, vitamin, and mineral elimination in the liver of animals infected with *Fasciola* and *Dicrocoelium* species.

**Table-4:** The macronutrients and mineral waste in livers infected with *Fasciola hepatica*, *Fasciola gigantica*, and *Dicrocoelium dendriticum* in abattoirs.

Macronutrients	Fasciolosis			Dicrocoeliosis		
	
	Sheep	Goat	Cattle	Sheep	Goat	Cattle
Water (g)	158,616	199405	297402	241026	201133	232788
Energy (kcal)	313,250	275310	567000	476000	397215	443813
Protein (g)	593.375	39192.3	85512	67762	56546.4	66933.5
Total lipid (fat) (g)	10851.9	9537.53	14112	16490	13760.7	11046
Carbohydrate, difference (g)	6,511,125	5722.52	16338	9894	8256.4	12788.4
Calcium (mg)	1118.75	9832.5	21000	17000	14186.3	16437.5
Iron (mg)	14,320	12585.6	20580	21760	18158.4	16108.8
Magnesium (mg)	4475	39330	75600	6800	56745	13150
Phosphorus (mg)	848,013	745304	1625400	1288600	1075318	59175
Potassium (mg)	689,150	605682	1314600	1047200	783873	1272263
Sodium (mg)	172,288	151421	289800	261800	218468	1028988
Zinc (mg)	26894.8	23637.3	16800	40868	34103.7	226838

**Table-5:** The level of Group B vitamin elimination in livers infected with *Fasciola hepatica, Fasciola gigantica*, and *Dicrocoelium dendriticum* at Arak abattoirs.

Vitamins	Fasciolosis			Dicrocoeliosis		
	
	Sheep	Goat	Cattle	Sheep	Goat	Cattle
Vitamin C (mg)	1566.25	1376.55	5460	2380	1986.075	4273.75
Thiamin (mg)	387.0875	338.675	793.8	588.2	488.6375	621.3375
Riboflavin (mg)	5459.5	4798.26	11571	8296	6922.89	9057.06225
Niacin (mg)	23605.625	20746.575	55325	35870	29932.9875	43312.8125
Vitamin B6 (mg)	2181.5625	1881.285	4548.6	3315	2714.3025	3560.3625
Folate, DFE (µg)	279687.5	245812.5	8000	425000	354656.25	9533750
Vitamin B-12 (µg)	133914.375	117695.025	249060	203490	169809.41	194948.75
Vitamin A, RAE Vitamin B-12 (µg)	523.21	23021815.5	20865600	39803800	33215685.75	16332300
Vitamin A, IU	87,387,800	76803624	70971600	132790400	110811636	55552175
Vitamin E (mg)	827.875	727.605	1596	1258	1049.7825	1249.25
Vitamin K (µg)	2013.75	2553.525	13020	3060	2837.25	10191.25

## Discussion

Growing populations and urbanization are translating into increased demand for livestock products, particularly in developing countries. Livestock has an essential role in socioeconomic and nutrition development. Global food security will require the production of more food using resources, including and more efficiently, and with less waste. Infectious disease is the main constraint of biologically efficient livestock production and both endemic and exotic disease results in mortality and morbidity and hence less food than should ideally be available in current farming systems [1,3,10]. Trematode infections occur worldwide, and widely differing effects on milk, meat, and fiber production and many new technologies have been developed to prevent or treat them. Approaches to developing better control of infection have included livestock breeding strategies, improved nutrition and management, and the development of new drugs, and diagnostic tests and vaccines [3].

Recently, human fasciolosis and dicrocoeliosis occurred occasionally but are now increasingly reported from entire the world. No continent is free from these diseases, and it is likely that where animal cases are reported, human cases also exist. The number of human cases affected by *Fasciola* spp. has increased drastically. Fasciolosis has many complications in humans. During the acute phase (caused by the migration of the immature fluke through the hepatic parenchyma), manifestations include fever, vomiting, diarrhea, abdominal pain, hepatomegaly, urticaria and eosinophilia, and can last for months. In the chronic phase (caused by the adult fluke within the bile ducts), the symptoms are more discrete and reflect intermittent biliary obstruction and inflammation. Occasionally, ectopic locations of infection (such as intestinal wall, lungs, subcutaneous tissue, and pharyngeal mucosa) can occur [34]. Different pathology studies in recent years proved that human infection is not only a serious health problem during the acute phase but also during the very long chronic period [35]. Most infections due to *D. dendriticum* infection in humans are light and asymptomatic. In heavier infections, symptoms may include abdominal pain, biliary colic, digestive disturbances, liver abscesses hepatomegaly, cirrhosis, cholecystitis, and seldom skin rash urticaria [36].

Based on the findings, fasciolosis and dicrocoeliosis are serious problems in the slaughterhouses of Arak, Iran, and impose great financial losses due to liver condemnation and weight loss. The prevalence of *Fasciola* and *Dicrocoelium* infections was 0.56% and 0.77%, respectively, which was relatively insignificant. Moreover, the prevalence of fasciolosis was 0.75% in sheep, 0.42% in goats, and 0.76% in cattle. Furthermore, the prevalence of dicrocoeliosis was 0.60% in goats, 1.14% in sheep, and 0.60% in cattle. The prevalence of liver fluke infections in ruminants depends on some factors such as the environment, the climate, and the choice of diagnostic methods. Changes in climatic conditions are closely correlated with changes in the prevalence of animal fasciolosis [37].

Obviously, husbandry conditions of the sheep (i.e., more contact with intermediate parasite hosts in comparison with cattle and goats) majorly contribute to the high incidence of diseases in sheep. Nevertheless, cattle and goats might show hereditary resistance. In the current research, fasciolosis (0.56%) was less prevalent in comparison with similar results which have been reported by Khoramian *et al*. [38] with 3.28%, Khosravi and Babbahamdy [39] with 8.48%, and Abdi *et al*. [40] with 0.98% in different parts of Iran. Our result is lower than these values.

Moreover, in the current research, the prevalence was lower than that found in Zimbabwe with 37% [41], in Kenya with 26% [42], in Zambia with 46% [43], in Southwest Zambia with 53.7% and 53.9% [44,45], and in Egypt with 14.7% [46].

In some regions of Iran, the prevalence of fasciolosis is low. Similar results have been reported in slaughterhouses in some regions of Iran. In this regard, Khoramian *et al*. [38] have reported similar findings in the present study. In their study, the prevalence of *Fasciola* infections was estimated at 3.28%, 2.76%, and 3.68% in sheep, goats, and cattle, respectively Khanjari *et al*. [7] reported slightly different results and showed an average prevalence of 6.6% for *Fasciola* infections in sheep and goats in the abattoirs of Amol in the north of Iran. Moreover, Khosravi and Babbahamdy [39] described a prevalence of 8.48% in the abattoirs of Ilam, Iran. The discrepancies in the prevalence of these infections might be attributed to significant differences between these studies in climatic conditions, including the average rainfall, temperature, seasonal changes, type of animals, and livestock management strategies [47]. Comparison of the present study with previous research indicated the reduced prevalence of liver fluke diseases in Iran, which might be associated with droughts, hindering disease transmission. Measures such as effective management of slaughtered animals have resulted in reduced prevalence of *Fasciola* and *Dicrocoelium* diseases; therefore, today, healthier animals enter the market. Two great features differentiate the epidemiology of *D. dendriticum* from *Fasciola* species. First, moist environment is not necessary for the intermediate hosts of *Dicrocoelium* species, which are widely present in meadows. Second, fluke eggs are able to survive on pastures for a long time [48]. The highest prevalence of *Fasciola* infections among all evaluated animals was reported during winter. This finding may be associated with meteorological evidence, which predicts many rainfalls during autumn and winter; furthermore, infection is quite prevalent in snails during rainy seasons. In general, it takes 12-16 weeks for a parasite to mature after infection; therefore, patent infection is predicted to be prevalent during winter [8].

The reduced rate of production, decreased growth rate of animals, and production of low-quality livestock products for humans, increased susceptibility to secondary infections, and cost of disease control measures are among the consequences of fluke infestation [49,50].

Furthermore, severe infestation might directly or indirectly lead to mortality through triggering or intensifying diseases. Today, scarce data are available regarding the presence of minerals and vitamins in the livers of infected animals; furthermore, the literature on this subject has not been reviewed so far.

The connection between economic loss and diseases, such as fasciolosis and dicrocoeliosis, is of great significance, especially regarding the nutritional elements removed from the humans’ nutrient cycle due to parasitic diseases. No records are available on the financial burden of fascioliasis and dicrocoeliosis in this region of Iran. Based on the findings, the prevalence and economic significance of fasciolosis and dicrocoeliosis were considerable in livestock slaughtered at Arak abattoirs.

However, in the present study, the annual ­economic loss was still moderately high. The high prevalence of infection is indicative of appropriate conditions for the growth and survival of parasites. The present findings highlight the importance of these diseases in farm animals and indicate the need for the development of control measures by studying the economic burdens and evaluating the local epidemiology of diseases. In the present study, the total annual economic loss was higher than that reported in Asella, Ethiopia [51]. Differences in the number of slaughtered animals in abattoirs and the average market price of liver and meat in different regions might account for the differences in economic loss in the studied regions.

The current findings showed that fasciolosis and dicrocoeliosis are still major risk factors for the health of livestock products in Arak, Iran. The financial loss resulting from liver condemnation and indirect weight loss was substantial in this study; ­therefore, adoption of control and prevention strategies is necessary.

## Conclusion

Fasciolosis and dicrocoeliosis were common and economically important infections in cattle, sheep, and goats at Arak slaughterhouses. Furthermore, the annual direct economic loss due to the condemnation of livers infested is relatively high. By studying the economic significance of these diseases and evaluating the local epidemiology, more practical and effective control strategies can be developed to prevent and limit the infection and reduce economic significant.

## Authors’ Contributions

MA designed, overseeing the research project, interpretation of data, and manuscript writing; EN collected the data, HH and MD helped in writing and review of the manuscript. All authors read and approved the final manuscript.
